# Page kidney: Rare cause of acute kidney injury after complicated renal artery angioplasty

**DOI:** 10.1111/jch.14318

**Published:** 2021-07-03

**Authors:** Adrianna Douvris, Januvi Jegatheswaran, Adnan Hadziomerovic, Marcel Ruzicka

**Affiliations:** ^1^ Division of Nephrology Department of Medicine The Ottawa Hospital University of Ottawa, Ottawa, Ontario, Canada; ^2^ Division of Radiology The Ottawa Hospital University of Ottawa, Ottawa, Ontario, Canada

**Keywords:** angioplasty, Page kidney, renal artery stenosis

## Abstract

The authors present a case of a patient who experienced a rare complication after attempted renal angioplasty and stenting, Page kidney. This patient presented with new onset hypertension secondary to bilateral renal artery stenosis and was referred for revascularization given hypertension refractory to medical management. The right renal artery underwent successful angioplasty and stenting; however, the left renal artery experienced recoil stenosis. Post‐procedure the patient developed acute kidney injury secondary to Page kidney from subcapsular and extracapsular hematoma. This was managed conservatively with transfusions and the hematoma and acute kidney injury self‐resolved over the next 4 months. This case highlights the importance of revascularization for refractory hypertension secondary to hemodynamically significant bilateral renal artery stenosis, the rare complication of Page kidney with attempted revascularization of renal artery stenosis and the involvement of a hypertension specialist in the decision of revascularization of renal artery stenosis.

## 1 BACKGROUND

Hemodynamically significant atherosclerotic renal artery stenosis causes hypertension. Opinion on treatment of choice has changed over the last 80 years since seminal reports on the pathophysiology of hypertension in response to unilateral and bilateral renal clips in dogs were published by Goldblatt and coworkers.^1^ Comprehensive medical therapy including large armamentarium of blood pressure lowering drugs, lipid lowering agents, and antiplatelet agents is endorsed as long as blood pressure is controlled, kidney function remains preserved, and patients do not develop acute pulmonary edema. This conceptual approach is based on results of clinical trials which showed no superiority f revascularizations by angioplasty and stenting compared to comprehensive medical therapy, and potentially significant risk of angioplasty and stenting related complications.^2^, ^3^ Here we report a rare but important complication of attempted renal artery angioplasty: unilateral perinephric hematoma described in the literature as Page kidney.

## 2 CASE PRESENTATION

A 69‐year‐old patient with a previous history of thoracic and abdominal aortic aneurysm was referred for assessment in the general nephrology clinic for new onset of hypertension. In parallel with the new onset of hypertension, her serum creatinine increased from normal (76 μmol/L) to 110 μmol/L and albumin to creatinine ratio from normal to 147 g/mol creatinine over the period of 6–12 months.

Her follow up CT angiogram post thoracic and abdominal aortic aneurysm repair showed new mild to moderate right renal artery stenosis and known left renal artery stenosis which progressed over the 3 years from mild to severe. The kidney sizes were identified as 8.9 and 8.1 cm for the right and left kidney, respectively.

In response to initiation of treatment with amlodipine 5 mg once daily and perindopril 2 mg once daily, her blood pressure improved and ranged from 124/70 to 150/80 mmHg, but creatinine increased from 110 to 155 μmol/L. Perindopril was discontinued at that time, and creatinine returned to baseline. Her blood pressure was however resistant to pharmacotherapy with amlodipine, bisoprolol, and clonidine. Attempts of restarting diuretic and/or angiotensin converting enzyme inhibitor were associated with immediate improvement in blood pressure control, but also with rapidly progressing acute kidney injury.

**FIGURE 1:**
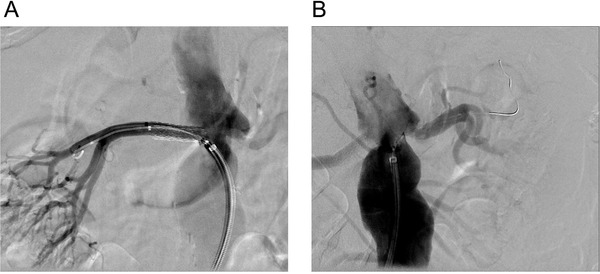
Successful angioplasty and stenting of the right renal artery stenosis (A) and unsuccessful angioplasty of the left renal artery stenosis (B)

The patient was reviewed by the vascular surgery team and interventional radiologists, and all agreed to proceed with bilateral renal artery angioplasty and stenting. Whereas angioplasty and stenting of the right renal artery stenosis was successful and uneventful (Figure 1A), angioplasty of the left renal artery stenosis, measured as an approximately 85% diameter reduction during the procedure, was complicated by post angioplasty recoil stenosis and unsuccessful attempts for stenting (Figure 1B).

**FIGURE 2:**
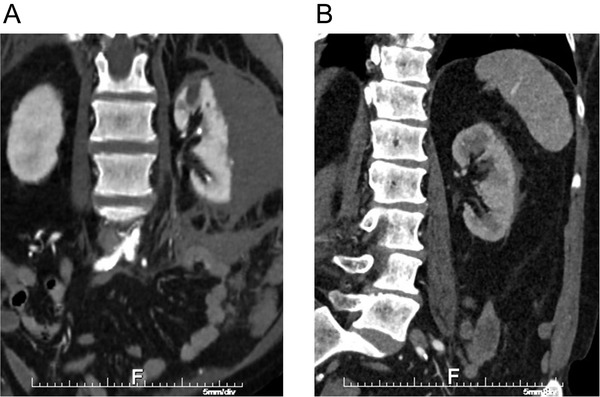
CT scan demonstrating large subcapsular and extracapsular hematomas of the left kidney post angiogram (A) and resolution of hematomas after 4 months (B)

Furthermore, the patient developed severe left flank pain, followed by brief hypotension within an hour after the procedure. Laboratory tests showed decrease in hemoglobin from 115 to 63 g/L and CT scan showed large left subcapsular and extracapsular hematoma (Figure 2A).

She received several transfusions of blood products. Her hemoglobin increased to 115 g/L and remained stable thereafter. Her blood pressure promptly increased to pre‐angioplasty levels and required reinstatement of amlodipine, bisoprolol, and clonidine. She had an acute kidney injury as assessed from an increase in serum creatinine to 150 μmol/L (from baseline of 110 μmol/L) within 12 h post procedure.

Over the following days to weeks, her creatinine decreased to baseline (100 μmol/L) and she tolerated addition of diuretic to her blood pressure lowering regimen to maintain her home blood pressure readings below 135/85 mmHg. Follow up CT angiogram in 4 months post procedure showed patent right renal artery with stent in place, moderate to severe stenosis of the tortuous left renal artery, and near complete resolution of the left perinephric hematoma with minimal residual at the lower pole of the left kidney (Figure 2B).

## 3 DISCUSSION

Page kidney or Page phenomenon is the result of subcapsular or extracapsular blood or fluid accumulation causing compression of the kidney parenchyma, resulting in decreased renal perfusion, activation of the renin‐angiotensin‐aldosterone system (RAAS), and hypertension.^4^ Subcapsular space is very limited, but Gerota's fascia could accommodate a significant amount of blood or fluid before causing renal parenchymal compression. Major sources of bleeding around the kidney can arise from trauma,^5^ medical procedures such as kidney biopsy^6^ or even be spontaneous due to tumors, cyst rupture, or vasculitis.^7^ Non‐bleeding causes of extracapsular compression include lymphocele, urinoma, etc.^4^

Page kidney following renal artery angioplasty has been rarely reported in the literature. There has been one reported case of subcapsular hematoma causing Page kidney following transplant renal artery stenting^8^ and a reported case of renal subcapsular hematoma complicating native renal artery stenting, the latter without mention of hypertension.^9^ In our case, attempted bilateral renal angioplasty was successful and uneventful in one kidney, but resulted in a large subcapsular and extracapsular hematoma in the other kidney, causing significant hemodynamic impairment and acute kidney injury. Successful angioplasty and stenting of the contralateral kidney prevented the undesirable outcome of hemodialysis and allowed hypertension to be controlled with blood pressure lowering drugs. Interestingly, subcapsular and extracapsular hematomas resolved within 4–6 months without need for surgical intervention. Our case demonstrates the importance of renal revascularization in the setting of hemodynamically significant bilateral renal artery stenosis causing hypertension refractory to comprehensive medical therapy. But this case also highlights the unpredictable and rare complication of attempted revascularization: Page kidney. Furthermore, our case supports that the decision for revascularization of renal artery stenosis should be taken by a specialist in hypertension after documented discussion with the patient including the risk of rare, but serious adverse outcomes.

## AUTHOR CONTRIBUTION

Marcel Ruzicka conceptualized and drafted the initial manuscript. Adrianna Douvris obtained patient consent to report the case. Adnan Hadziomerovic provided the images for the manuscript, and important input into the technical aspects of the case. Adrianna Douvris, Januvi Jegatheswaran, and Marcel Ruzicka all contributed to manuscript review and editing. Adrianna Douvris, Januvi Jegatheswaran, Adnan Hadziomerovic, and Marcel Ruzicka all approved the final version. Marcel Ruzicka is the guarantor of the manuscript.
